# Portable Wind Energy Harvesters for Low-Power Applications: A Survey

**DOI:** 10.3390/s16071101

**Published:** 2016-07-16

**Authors:** Seyedfakhreddin Nabavi, Lihong Zhang

**Affiliations:** Department of Electrical and Computer Engineering, Faculty of Engineering and Applied Science, Memorial University of Newfoundland, St. John’s, NL A1B 3X9, Canada; lzhang@mun.ca

**Keywords:** wind energy harvesting, piezoelectric, electromagnetic, electrostatic, rotational, aeroelastic, macro-scale, micro-scale

## Abstract

Energy harvesting has become an increasingly important topic thanks to the advantages in renewability and environmental friendliness. In this paper, a comprehensive study on contemporary portable wind energy harvesters has been conducted. The electrical power generation methods of portable wind energy harvesters are surveyed in three major groups, piezoelectric-, electromagnetic-, and electrostatic-based generators. The paper also takes another view of this area by gauging the required mechanisms for trapping wind flow from ambient environment. In this regard, rotational and aeroelastic mechanisms are analyzed for the portable wind energy harvesting devices. The comparison between both mechanisms shows that the aeroelastic mechanism has promising potential in producing an energy harvester in smaller scale although how to maintain the resonator perpendicular to wind flow for collecting the maximum vibration is still a major challenge to overcome for this mechanism. Furthermore, this paper categorizes the previously published portable wind energy harvesters to macro and micro scales in terms of their physical dimensions. The power management systems are also surveyed to explore the possibility of improving energy conversion efficiency. Finally some insights and research trends are pointed out based on an overall analysis of the previously published works along the historical timeline.

## 1. Introduction

Energy harvesting has become an interesting field of research in recent few years with a target of meeting energy requirements for low-power electronic applications, such as implantable biosensors [1,2], consumer electronics [3], military equipment [4], and wireless sensor networks (WSN) [5,6]. Normally only batteries are used to power the aforementioned devices that feature low power consumption. However, size limitation and recharging necessity prevent a capability of autonomy. Due to these concerns, more efforts are striving to replace the batteries with more efficient power solutions that have no lifetime worries. The energy harvesting from ambient resources is the key of this technology.

Portability, as an important factor in many practical applications, is strongly demanded. The level of portability is identified by device dimension and lightness. High portability promises easy deployment and reduced interfacing cost. The micromachining technology is known as one major method for producing portable devices. By using lithography and etching techniques to expose the designed patterns on silicon surface, this technology can manufacture micrometer-sized or even smaller mechanical parts. Several processing methods for micromachining fabrication, such as bulk micromachining [7], surface micromachining [8], and LIGA (Lithographie, Galvanoformung, Abformung) [9], have been proposed. Micromachining has been interpenetrating several disciplines (e.g., medicine, optics, mechanical and electrical engineering). Likewise, integrated circuits (IC) can be fabricated with similar procedures. Advanced design automation methods can enhance the fabrication quality and circuit performance by considering the electronic device parasitic effects [10] and manufacturability issues [11]. Due to similarity in the fabrication, micromachining and microelectronics can be integrated to offer micro-electro-mechanical-system (MEMS), a practical micro-system with high portability and reliability [12].

The available natural harvestable energy in outdoor environment includes solar, thermal and vibrations. Solar power system is one of the most usual methods of energy harvesting [13]. Since high solar energy conversion efficiency is highly dependent on surface area of solar cells in addition to availability of sunlight, MEMS or other portable devices, which may not gain the best profit from solar energy due to their inherent tiny physical size, require other ambient energy sources. Electricity generation associated with thermal is another approach for MEMS or other portable energy harvesters. The temperature difference between two various materials can offer electrical power, for example thermocouples [14]. However, large temperature gradient is not feasible for the MEMS or other portable devices with miniature sizes [15].

Vibrations are known as an attractive source of harvestable energy thanks to ubiquity in the environment and non-complex structure for trapping them [16]. Wind flow is a common vibration source, which is widely and naturally accessible. The induced vibration by wind is dependent on wind flow velocity [17,18]. In addition, fluid can be considered as another ambient vibration source, which can be utilized in different areas such as river currents, tide motion, and ocean’s waves [19,20,21].

Table 1 presents ambient harvestable energy sources including solar, wind, thermal, and fluid (i.e., water drop) [22,23]. As an important factor of expressing power conversion capability, power density is also listed in Table 1 for evaluation purpose. The comparison among power densities of ambient energy sources shows that solar cells have the highest power conversion potential when sunshine is abundant. Nevertheless, it may deliver unsatisfactory performance in some applications due to lack of sufficient sunlight [24]. Furthermore, because of low power conversion efficiency in thermal and fluid flow sources as well as difficulty in fabrication, they are not highly qualified for popular energy harvesting. Under such a condition, wind flow may be considered as one promising candidate. A successful example of wind energy harvesting is wind farms. The large-scale wind turbines, which have been known for centuries, can generate kilowatts or even higher electrical power for on-grid systems or provide wind power plants [25]. Since last decade, some research has been conducted on small-scale wind energy harvesting [26]. But it is still far from being commercially available.

This paper is mainly focused on portable wind energy harvesters for a target of low power consumption applications. In Section 2 we will present different portable wind energy harvesting techniques. Section 3 will categorize the portable wind energy harvesters based on the applied mechanisms. In Section 4, wind energy harvesting devices are classified in terms of geometry dimensions. The power management systems, which make the portable energy harvesting devices usable in real applications, are discussed in Section 5. Finally Section 6 will give a conclusion on the survey as well as some insights in this area.

## 2. Agile Wind Energy Harvesting Techniques

The generation of milliwatt electrical power from wind flow energy is relatively new compared to the conventional power generating techniques. The wind harvesting devices normally consist of fixed components and active parts, which are vibrated by mechanical power of wind flow. Scalable in size, these energy harvesters can offer milliwatt output power. The quantity of generated power depends on system size and wind flow velocity in the ambient environment. Theoretically the mechanical power available from a cross sectional area between wind flow and energy harvesting devices can be expressed by [27]:

(1) $$P=\frac{1}{2}A\rho V^{3}$$

where *A* is the cross sectional area in square meters (m^2^), ρ is the density of flow that might be air or water density in the unit of kilogram per cubic meter (kg/m^3^), and *V* is the velocity of the flow in meter per second (m/s). Albert Betz concluded that a wind turbine theoretically cannot convert more than 59% of the kinetic energy of wind to mechanical energy, which is known as Betz limitation [28]. Considering this limitation, power coefficient (*C_p_*) and efficiency (*η_g_*) of a generator can be inserted into Equation (1), which can be rewritten as follows:

(2) $$P=\frac{1}{2}{A\rho V}^{3}C_{p}\eta_{g}$$

To realize the energy conversion modeled in Equation (2), the following three main steps during electric power generation should be completed by an energy harvester:

(1) Collect the mechanical stresses from ambient sources, which are applied on the mechanical active part of the harvester;
(2) The kinetic energy offered by wind as vibration sources is converted to electrical energy;
(3) Process the generated power to make it as DC voltage and store it in a super-capacitor cell.

In this paper, Section 2 and Section 3 are aimed to address Steps (1) and (2) by reviewing the energy harvesting devices published thus far, while Section 5 will discuss the tips of Step (3) above.

A variety of performance metrics have been utilized for evaluating the capability of energy harvesting in terms of energy conversion from mechanical to electrical power. Some of these figures of Merits (FOMs), such as charge constant (*d*) and voltage constant (*g*), are only related to piezoelectric materials, while the geometry size of energy harvesters is not taken into account [29]. To include more factors, Aktakka et al. [30] proposed normalized power density times bandwidth as a FOM. Although this metric indeed is a comprehensive FOM, most of the published papers in the literature provide insufficient information about bandwidth that their proposed devices can achieve. A straightforward multiplication between normalized power density and bandwidth may fail to provide a comparison for the largest pool of the existing harvesters. Therefore, in this paper we use the normalized power density, which is defined below, as one FOM:

(3) $$P_{\mathrm{density}}=\frac{P_{m}}{\mathrm{Volume}\;\times \;V}\;$$

where P*_m_* is the measured electrical peak power, Volume is the volume of the harvester, and V represents the wind speed. Moreover, the power coefficient $C_{p}$, which is more relevant in the context of non-linear wind velocity with reference to the amount of harvested power, is considered as another FOM in this paper. It can be defined as [26]:

(4) $$C_{p}=\frac{P_{m}}{0.5\times \rho_{\mathrm{air}}\times A\times V^{3\;}}\;$$

To convert ambient vibration (including wind energy) to electricity, piezoelectric- [24], electromagnetic- [31], and electrostatic-based [32] techniques have been introduced in the previous research. Among them, each technique has its distinct advantages as well as disadvantages. The electrostatic-based technique can build up a tiny energy harvester if using micromachining process. Therefore, this technique is quite useful for MEMS-based energy harvesters. Certain analytic modeling methods [33] can be used to formulate the coupling and substrate capacitance in the micrometer or nanometer technologies. However, to function well, such energy harvesters need to manage a large initial voltage (as bias voltage) or electret [34] for supplying electrical charges between capacitor plates. In contrast, the piezoelectric- and electromagnetic-based techniques, which are more often used for portable wind energy harvesting, need no external power sources.

Theoretically the power produced by the piezoelectric-based technique can be expressed by Equation (5):

(5) $$\frac{1}{2}\times \sigma^{2}\times \frac{K^{2}}{2c}$$

where σ is the applied stress, K is the coupling coefficient, c is the elastic constant. As shown in the analytic equation above, amount of the harvested power is dependent on several parameters, such as the applied stress on piezoelectric materials provided from ambient vibrations, the coupling coefficient and the elastic constant. Both the coupling coefficient and elastic constant are related to piezoelectric material properties. It is obvious that enhancing the applied stress on piezoelectric materials can produce more energy [35].

Similarly, power generation with the electromagnetic-based technique can be theoretically estimated by Equation (6):

(6) $$\frac{1}{2}\times \frac{\;B^{2}}{\mu_{0}}$$

where *B* is the magnetic field and *μ_0_* is the magnetic permeability constant of free space. Since there is a non-linear relationship between the generated power and electromagnetic field, enhancing the magnetic field strength is one effective way for increasing the produced power [36]. Arroyo et al. [37] investigated both piezoelectric- and electromagnetic-based techniques theoretically and experimentally. According to the provided information in this study, the piezoelectric generators have low coupling coefficient and low loss coefficient, while the electromagnetic generators have high coupling coefficient and high resistive loss coefficient. Furthermore, the study of scaling effect in this work shows that, different from the commonly accepted hypothesis, the power density of the electromagnetic-based generators does not necessarily decrease in proportion to their volumes.

Amount of the produced power by the electrostatic-based generators can be calculated by Equation (7):

(7) $$\frac{1}{2}\times \varepsilon_{0}\times E^{2}$$

where *ε_0_* is the electrical permittivity of free space, and *E* is the electric field. Therefore, magnitude of the generated power is strongly determined by the strength of the electric field, which is in turn up to the magnitude of bias voltage or electret [38]. The comparison between electrostatic- and piezoelectric-based generators, as presented by Elliot et al. in [39], shows that the electrostatic-based technique performs better than the piezoelectric-based technique at low acceleration scenarios due to lower energy losses, while the piezoelectric-based technique outperforms the electrostatic-based technique at high acceleration operating conditions. However, the MEMS scale piezoelectric-based energy harvesters at very high accelerations may have lower performance due to the dielectric breakdown limit of piezoelectric materials. Thus, the electrostatic-based technique is preferred for MEMS energy harvesters if a very high acceleration operating condition is expected. In the following sub-sections, each power generation technique will be discussed in more detail.

### 2.1. Piezoelectric-Based Generators 

Due to dichotomy behavior, piezoelectric materials have been utilized in many types of sensors and actuators [40,41]. There are different natural crystal classes capable of piezoelectricity, among which three are more commonly utilized in micromechanical systems. They include zinc oxide (ZnO) as a MEMS transducers [42], aluminum nitride (AlN) for thin film depositing [43], and $\mathrm{Pb}({\mathrm{Zr}}_{x}{\mathrm{Ti}}_{1-x}$)$O_{3}$ (PZT) as an active material [44]. Among them, PZT is the most popular option for power conversion due to its high electromechanical coupling factor as defined below:

(8) $$K^{2}=\frac{\text{Electrical Energy converted}}{\text{Mechanical energy applied}}≅\frac{{\left(\frac{d_{31}}{s_{11}}\right)}^{2}}{\varepsilon_{33}}$$

where K is the electromechanical coupling factor, d_31_ is the piezoelectric constant, $\varepsilon_{33}$ is the dielectric constant, and $s_{11}$ is the mechanical compliance coefficient. For a better comparison, the relative electromechanical coupling factors for three major types of piezoelectric materials are listed in Table 2 [45]. We can see that PZT has more than double electromechanical coupling factor over AIN or ZnO. Therefore, this type of piezoelectric materials has been widely used for power generation in portable energy harvesting devices [18].

**Figure 1:**
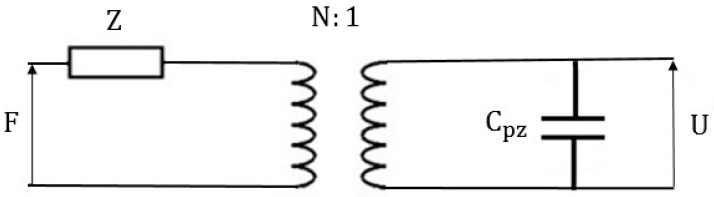
Equivalent circuit of the piezoelectric-based generators.

The equivalent circuit of piezoelectric generators, which expresses both mechanical and electrical parts, is depicted in Figure 1 [46]. At input of this circuitry, external excitation force *F* is applied. The mechanical branch has one resonant impedance element (*Z*), which is dependent on stiffness of mechanical resonator and mechanical branch mass. The transformation ratio is equal to *N = C_pz_h_33_*, where *C_pz_* is the clamped capacitance of piezoelectric material mainly measured when piezoelectric layer is not deformed and *h_33_* is the ratio between piezoelectric coefficient and mechanical compliance. On the right side, the transformer and clamped capacitance illustrate power conversion in energy harvesters with *U* (voltage) presented as output [47].

For the first time, Carroll introduced a small energy harvester for collecting energy from water flow based on piezoelectric material [27]. In comparison with the conventional energy harvesters such as wind turbines [48], its size, which was significantly reduced, can offer great portability to a variety of applications. Equation (2) theoretically and the experimental data from [27] empirically show that the amount of the generated electrical power is highly dependent on harvester dimension and flow speed. In parallel with size reduction for a goal of increasing portability, drop of the output power amplitude is prevalent. To overcome this issue, some research has been conducted to apply different structures or materials to magnify the conversion results [49,50].

### 2.2. Electromagnetic-Based Generators 

The amount of induced voltage in the electromagnetic coil can be computed by [51]:

(9) $$U=-N\frac{d\phi }{dt}$$

where *U* is the generated voltage, *N* is the number of turns, and ϕ is the total flux linkage. The flux density is highly dependent on magnetic field, which is given by:

(10) ϕ=BA sinθ

where *B* is the magnetic field, *A* denotes the perpendicular area to the magnetic field, and *θ* is the angle at which the magnetic field contacts the coil.

**Figure 2:**
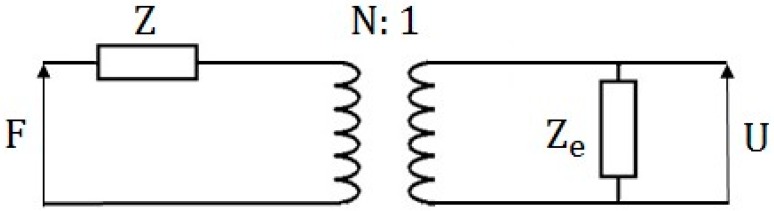
Equivalent circuit of the electromagnetic-based generators.

The equivalent circuit for explaining the working principle of the electromagnetic-based generators is displayed in Figure 2 [52]. Similar to the piezoelectric equivalent circuit, a mechanical force *F* as input is applied to a mechanical resonator with resonant impedance *(Z)*. A transformer can convert mechanical power to electrical power with ratio *N* following *N = BL/Z_e_*, where *L* is the length of the wire that constitutes the coil and *Z_e_* is the coil impedance. The coil impedance is relevant to the inductance and resistance of the coil [47]. The conversion result from mechanical energy to electricity is depicted by *U* (voltage) in Figure 2.

**Figure 3:**
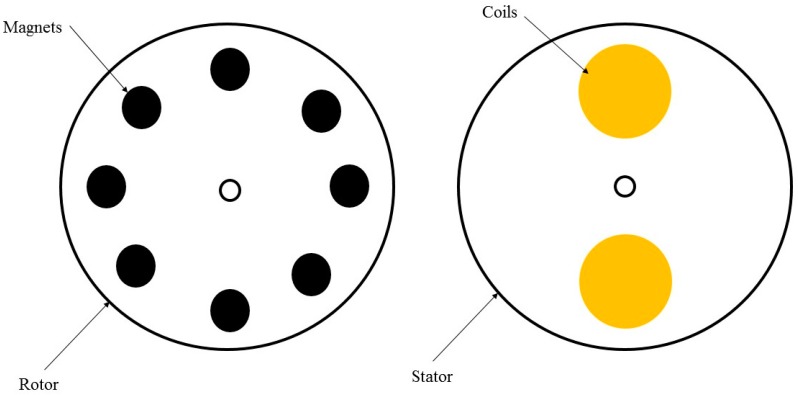
Rotor and stator for the axial-flux alternator.

The interaction between wind flow and moveable part of an electromagnetic energy harvester can provide relative motion, which is bound to produce electrical power. Based on the flux density change in proportion to the conductor location change, several wind flow energy harvesters have been devised. Weimer et al. [53] proposed a circular electromagnetic wind energy harvester for the axial-flux alternator, where the rotor included 8 neodymium magnets in an alternating pole configuration (as shown in Figure 3) and the stator was manufactured with two separate copper wire coils with 1250 turns. The diameter of the stator and rotor plates is 76.2 mm. The wind flow can rotate the rotor plate, which induces electrical voltage in copper wires. The maximum harvested electrical power in this study at the highest wind speed (8.94 m/s) is 651 μW. 

**Figure 4:**
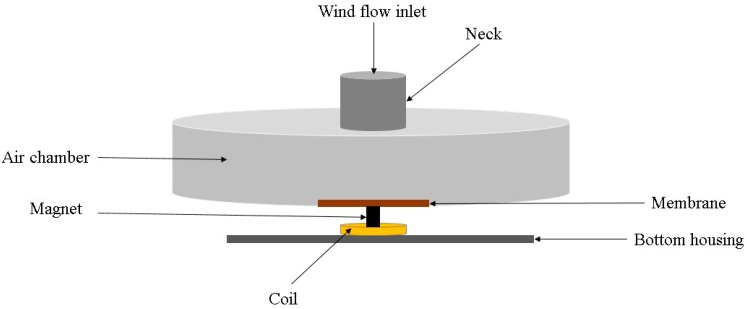
Schematic diagram of the Helmholtz resonator-based energy scavenger.

A Helmholtz-resonator-based electromagnetic energy scavenger was introduced by Kim et al. [54]. The simplified schematic of the proposed Helmholtz resonator-based energy harvesting is depicted in Figure 4. This Helmholtz resonator has a chamber, which is simply filled with gas (air), with an open neck in the middle. The air inside the chamber has spring behavior and the air inside the neck acts as mass. Thus, this system can be modeled by a second-order differential equation. To increase the efficiency, a diaphragm (membrane) is attached to the bottom wall of the resonator with a magnet fixed on this diaphragm. While fluidic oscillation happens for the diaphragm due to mechanical energy of wind flow, the magnet attached to the diaphragm vertically vibrates inside the coil. This devised energy harvester includes two parts, a cylindrical chamber with 19 mm diameter and 5 mm height, a neck on top of the chamber with 3 mm diameter and 5 mm height. It could produce peak to peak voltage output of 4 mV at 5 m/s wind velocity. However, the power conversion efficiency of the proposed wind energy harvester was quite low even at high wind speed. Thus, it may not function very well for practical applications.

**Figure 5:**
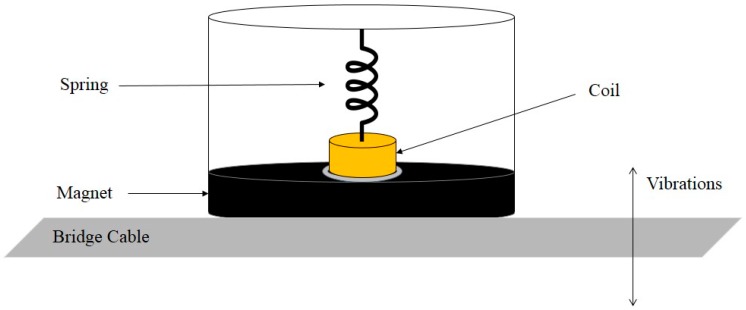
Schematic diagram of the wind-induced energy harvesting system.

Another configuration that can function well is to use coils as moveable component and consider magnets as the fixed part of electromagnetic generators. Feasibility of an energy harvesting system based on wind-induced vibration of bridge cables was investigated by Jung at al. [55]. In this study, an electromagnetic-based generator was introduced to harvest wind-induced vibration energy due to interaction between wind flow and cables. The proposed device, as depicted in Figure 5, includes a big circular magnet with an opening in the center. The proposed energy harvester has a suspended coil, which is connected to a spring. The ambient vibrations cause oscillation to the coil, which can induce the relative motion inside the magnet. In comparison with the other research work, the proposed device in this study can provide greater magnetic flux due to use of a big magnet compound (enclosing the fixed magnet and moving coil). With 80 mm diameter and 10 mm thickness, the utilized circular magnet could generate magnetic flux density of 0.5 Tesla. The experimental results showed that this device could generate 27.14 mW RMS power at 5.4 m/s wind speed.

By comparing the existent piezoelectric and electromagnetic techniques above, we can see the presented equivalent circuits for both approaches are almost the same. As illustrated in Figure 1 and Figure 2, the output impedance is capacitive for the piezoelectric generators and resistive for the electromagnetic generators. For adapting load impedance, the electromagnetic-based generators need a load impedance of some kilo ohm, whereas the piezoelectric-based generators require a load impedance in mega ohm range. Furthermore, the damping factor for the piezoelectric generators is constant because the used piezoelectric material has unchangeable surroundings. However, the electromagnetic-based generators have varied damping factor, which may change along with electromagnetic or resistive load variation. Furthermore, a minimum deformation in the electromagnetic-based generators has to be required, which means the wind flow should be strong enough to provide such a minimum deflection.

### 2.3. Electrostatic-Based Generators 

**Figure 6:**
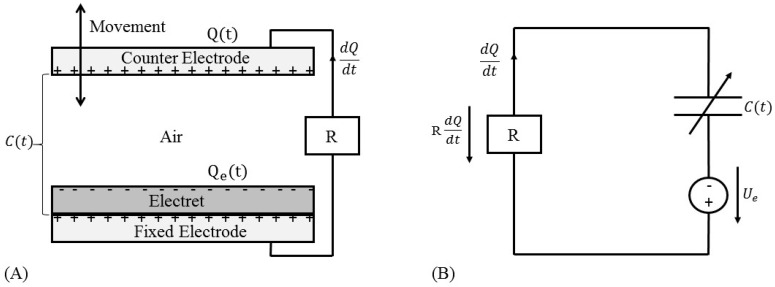
(**A**) Schematic view of the electret-based electrostatic energy harvesters and (**B**) equivalent circuit.

Another technique for electrical power generation from ambient environment is electrostatic method, whose operation is based on a capacitive structure created by two standalone electrodes. The gap between both electrodes can be filled with air, vacuum, or any dielectric materials [56]. The variation of capacitance, which takes place by moving one of the electrodes, is able to convert mechanical vibration to electricity by charge-discharge cycles or electret. This type of generators can be categorized into two groups [57]: (1) Electret-free electrostatic generators, whose functionality is dependent on external bias voltage [32]; (2) Electret-based electrostatic generators, which are able to directly turn ambient vibration to electricity thanks to electret utilization on the surface of one or both electrodes. Electrets are the dielectric material with a capability of keeping electric field and surface potential inside the structure for years just like the magnets in the electromagnetic-based generators [58]. Figure 6 illustrates the electret-based generator and its equivalent circuit, where $Q_{e}$ is the electret charge, Q is the sum charge between counter (movable) electrode and fixed electrode, *R* is the load resistance, C(t) is the capacitance of the electrostatic energy harvester, and $U_{e}$ is the surface voltage on electret [59]. The relative motion of the counter electrode leads to variable capacitance, which induces electrical current through load.

**Figure 7:**
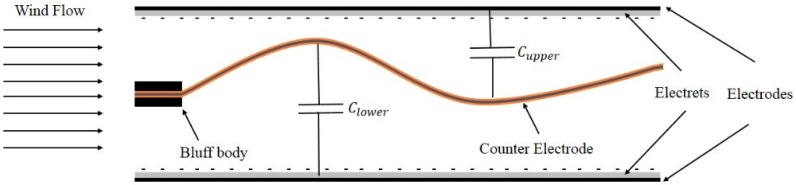
Schematic representation of the proposed wind energy harvester in [59].

If the prerequisite initial electrical charge for capacitor polarization is realized by the electret materials, promising harvesting performance may be offered by the electret-based electrostatic energy harvesters. Recently Perez et al. [59] introduced a wind energy harvester by deploying the electret-based electrostatic technique. The proposed device in this study includes two parallel copper plates with the size of 50 mm and 200 μm in length and thickness, respectively. Both copper films are covered with a 25 μm Teflon PTFE layer, which was fabricated by corona discharge. Furthermore, a membrane with 25 μm thickness of PVDF, whose two sides were deposited with 10 nm gold layer, is attached to a bluff body. The schematic diagram of this wind energy harvester is illustrated in Figure 7. The proposed system has two capacitors between the membrane and upper electrode ($C_{upper}$) as well as lower electrode ($C_{lower}$). When wind flow blows the membrane to move upwards and downwards, capacitance of the energy harvester would be changed continuously. When wind flow velocity reaches 10 m/s, the membrane can be oscillated. The average output power during period time *T* is proportional to the capacitance variation between $C_{max}$ and $C_{min}$, which can be determined by:

(11) $$P_{elec}=\frac{1}{T}\int_{0}^{T}R{\left(\frac{dQ}{dt}\right)}^{2}dt\propto \left(C_{max}-C_{min}\right)U_{elec}^{2}f$$

where $U_{elec}$ is the surface electret voltage, f is the osculation frequency, $C_{max}$ and $C_{min}$ are maximum and minimum possible capacitances, respectively. 

The experimental data in this study show the proposed wind energy harvester was able to produce 2.1 mW power and 200 V output voltage, when the electret voltage is −650 V with 30 m/s wind speed. Even though this wind energy harvester has a simple structure, the required high wind speed (10 m/s) for fluttering the membrane makes it somehow impractical for the regular windy conditions. In addition, a special power management system has to be considered due to the generated high output voltage and low current.

## 3. Portable Wind Energy Harvesting Mechanical Mechanisms

The energy harvesting devices need to function as a mechanical resonator for converting wind flow to vibrations. The direct reaction between wind flow and moveable part of energy harvesters is oscillation. This periodic oscillation can affect the active part of the energy harvesters. All the portable wind energy harvesters in the literature follow two distinct mechanical mechanisms for trapping wind flow vibrations. In this regard, we can classify the wind energy harvesters into two groups: rotational and aeroelastic harvesters. Below each group is discussed in more detail.

### 3.1. Rotational Harvesters

**Figure 8:**
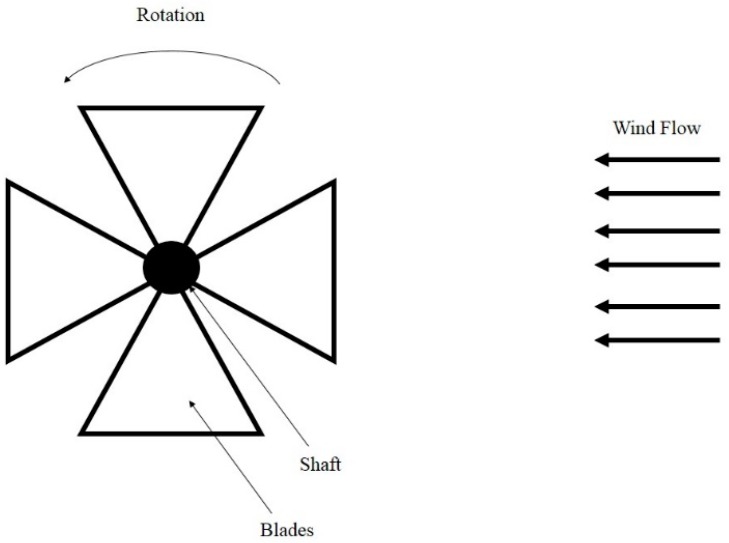
Diagram of portable wind energy harvesting devices based on rotational mechanism.

Figure 8 shows the mechanical structure of rotational wind energy harvesters. The rotational movement is a circular motion of an object with reference to its center. This type of motion has been widely used in the traditional large-scale windmills [60]. Due to high reliability and accumulated mature design knowledge, this mechanism has been widely used for small-scale wind energy harvesting devices [26]. Normally, the rotational-mechanism-based devices, such as the one in [61], may include a fan with multiple blades and a jamb, which is fixed in the centre of the fan. The energy of wind flow can rotate the fan around the fixture. The intensity of rotation is related to wind flow speed. In other words, wind blowing in higher speed can induce circular motion with higher frequency of rotation for the moveable part of the harvesters.

**Figure 9:**
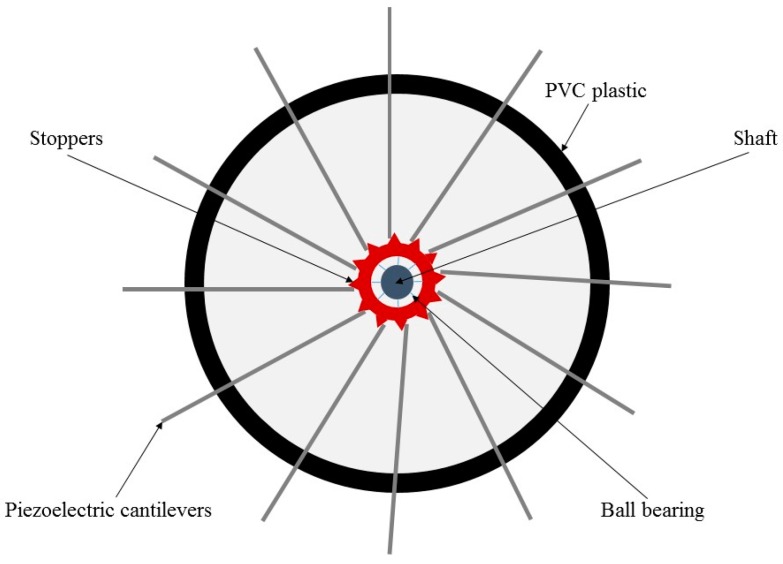
Schematic diagram of the piezoelectric windmill consisting of 12 piezoelectric cantilevers.

The rotational mechanism has been used for piezoelectric-, electromagnetic- and electrostatic-based wind energy harvesters. In the piezoelectric-based harvesters, one side of the piezoelectric cantilever is clamped by the fan, and the other side is free. Then the induced rotation in the fan can move the piezoelectric cantilever. The interaction between the shaft, which is a fixed point in the center of the fan, and the moveable side of the piezoelectric cantilever applies relative stress to the piezoelectric material. Priya et al. [62] introduced a small-scale wind energy harvester based on piezoelectric direct effect, which was combined with the traditional windmill features and piezoelectric properties. The proposed device included 12 bimorph piezo-cantilevers, which were attached to a quite small fan. The schematic of such a piezoelectric windmill with 12 cantilevers is illustrated in Figure 9. Wind flow could apply oscillatory stress on the cantilevers since the fan could be easily rotated at the normal wind flow conditions. The dimension of each individual bimorph piezoelectric cantilever was 60 × 20 × 0.6 mm^3^. This energy harvesting device was able to generate power of 10.2 mW at the frequency of 6 Hz.

Another rotational piezoelectric wind energy harvester, which was introduced by Yang et al. [63], consisted of 12 micro-cantilevers with 47 × 20 × 0.5 mm^3^ dimension behind the fan. One or more than one shaft (ball) was located at the center of the fan. Wind flow could rotate the fan, while the interaction between shafts would apply relative stress to piezoelectric cantilevers. The prototyped device in this research could generate 613 μW rectified power. In comparison with the conventional energy harvesters such as wind turbines, the size of the developed devices in these studies was significantly reduced. In spite of the size reduction, how to prevent reducing the harvested power is a challenging question to be addressed.

The frequency of the applied stress is proportional to the rotational frequency of the fan. The advantage of the piezoelectric-based harvesters is the huge stress applied to the cantilevers at the center of the fan due to high torque at that location, which can be computed by *τ* = *Fd*, where *F* is the excitation force (wind flow) and *d* is the distance between the force and the free end of the cantilever. Consequently, the maximum torque may appear around the center of the rotational structure, where shaft is located. Furthermore, increasing the number of the piezoelectric cantilevers may increase the number of stoppers, which are actually the confluence nodes between cantilevers and shaft. As a result, the rotational frequency would be dropped. Therefore, higher wind flow speed is required for the proper function of these harvesters.

On the other side, for the electromagnetic-based harvesters, the required movement for the rotor can be achieved by the mechanical rotational mechanism, which is able to provide a relative motion between the rotor and stator in the harvesters [53]. The rotational structure normally encloses a big plane, which can provide sufficient space for locating magnets or accessing coils on the top. This available space can also allow the designers to enlarge the generated power by adding more magnets. But it should be noted that, by applying more magnets for enhancing magnetic field, the mechanical rotational structure might gain a lot of weight, which in turn requires stronger wind flow to make rotation feasible. Similarly, the rotational mechanism can be used by the wind energy harvesters based on electrostatic generators. In a recent study conducted by Perez et al. [64], a small wind turbine with 4 blades within 4 cm rotor diameter to extract power from wind flow was devised. The proposed device could generate 1.8 mW output power at 10 m/s wind speed. The main advantage of this energy harvester is its capability of generating 95 μW power at wind speed as low as 1.5 m/s.

The main advantage of the rotational mechanism is to provide an alternative and strong spin with sufficient space for patterning the active part of an energy harvester. But this mechanism needs a relatively larger space for making rotation viable. Therefore, the portability associated with this mechanism is not high. For this reason, no MEMS wind energy harvester entirely based on rotational mechanism has been developed thus far.

### 3.2. Aeroelastic Harvesters

**Figure 10:**
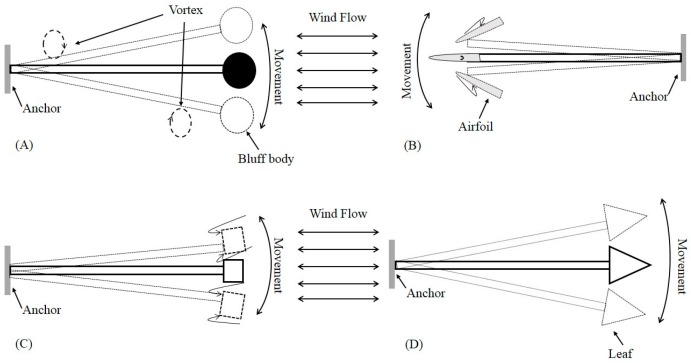
Common aeroelastic mechanisms for wind energy harvesting: (**A**) vortex shedding, (**B**) flutter, (**C**) galloping and (**D**) flapping leaf.

When a mechanical resonator is immersed in a fluid flow, aerodynamic phenomena (such as vortex shedding, fluttering, and galloping) may appear around or on the structure. As a result, vibration can be observed as shown in Figure 10, which presents the most common aerodynamic mechanisms, Figure 10A vortex shedding with a bluff body, Figure 10B flutter with attachment of an airfoil, Figure 10C galloping with attachment of a prismatic object, and Figure 10D flapping leaf [65,66].

The existing aeroelastic mechanisms in the literature for wind energy harvesting can be categorized into two groups: (1) vortex induced vibrations (VIV) and (2) movement induced vibrations [26,67]. Normally in VIV, a cylindrical bluff body is located at the end of cantilever in a wind energy harvester. When wind passes the bluff body, the resulting flow would form discrete vortices due to gradient pressure. The pattern of the vortices shedding alternately from one side of the body and then the other side is called Karman vortex street. In the situations where vortex shedding behavior is periodic, the frequency can be determined by [26]:

(12) $$f=\frac{SV}{D}$$

where *V* is the wind flow speed, *D* is the characteristic dimension (e.g., the diameter if a circular cylinder is used as a bluff body or the channel diameter if a fluid channel is used), and *S* is the Strouhal number that is a dimensionless number for describing oscillating flow mechanism. The Strouhal number depends on both the body shape and the Reynolds number, which may be calculated with Equation (13):

(13) $$Re=\frac{DV\rho }{\mu }$$

where ρ is the flow density, and μ is the flow viscosity. When the vortex shedding frequency is close to one of the harmonic natural frequencies of the aeroelastic harvester, lock-in or synchronization takes place so that the energy harvesting maximum power is feasible under this condition [68].

**Figure 11:**
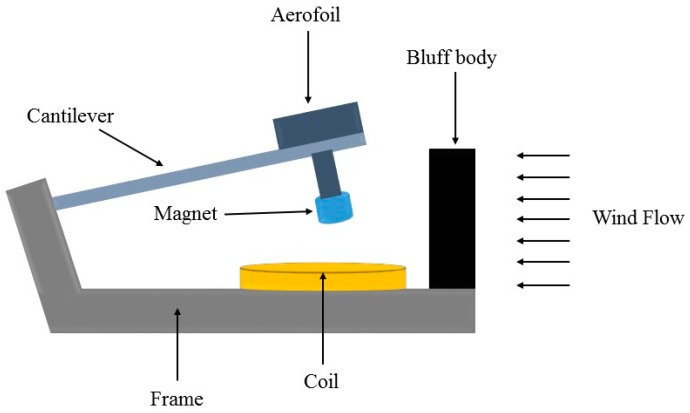
Schematic diagram of the electromagnetic wind energy harvester based on VIV mechanism.

Zhu et al. [69] presented an electromagnetic wind energy harvester based on VIV. The proposed device includes a 50 mm × 18 mm × 0.2 mm cantilever made of Beryllium Copper (BeCu). As shown in Figure 11, a cylindrical magnet, which is made of NdFeB-38H with 15 mm diameter and 10 mm height, is fixed at the end of the cantilever with certain distance (15 mm) from an 80 mm × 25 mm × 6 mm rectangular aerofoil, which is located at the other side of the cantilever. The overall size of the device is 12 cm × 8 cm × 6.5 cm. According to reported experimental data, the proposed electromagnetic generator could operate with harvested electrical output power of 470 μW at the wind speed as low as 2.5 m/s. When the wind speed reached 5 m/s, the amount of output power was 1.6 mW. Although the harvested electrical power by the proposed device in this study is enough for most of low power electronic devices, using bluff body may somehow reduce level of portability. In addition, high cut-in wind speed (2.5 m/s) is another drawback of the proposed energy harvester.

The VIV mechanism is also capable of being utilized in the energy harvesters based on piezoelectric generation technique [70]. In this type of wind energy harvesters, a cantilever beam is normally fixed on one end and the other end can move freely. Piezoelectric material is deposited in an area close to the root of the cantilever. A lumped-parameter model for describing the coupling behavior of the piezoelectric VIV-based wind energy harvesters can be written as [71]:

(14) $$M_{eff}\ddot{w}+C\dot{w}+Kw+\Theta U=F_{L}$$

(15) $$C_{pz}\dot{U}+\frac{U}{R}-\Theta \dot{w}=0$$

where *w* is the displacement of the cantilever tip, $M_{eff}$ is the effective mass, *C* and *K* are the damping coefficient and stiffness respectively, Θ is the electromechanical coupling coefficient, *U* is the generated voltage, $C_{pz}$ is the piezoelectric material capacitance, *R* is the load resistance, and $F_{L}$ is the vortex induced force.

As the second group of aeroelastic mechanism for wind flow trapping, movement-induced-vibration has a wider scope than the first VIV group, including (i) flutter-based; (ii) galloping-based; and (iii) flapping-leaf-based harvesters. This type of aeroelastic mechanisms is affected directly from wind flow force. As a result, movement appears right on the fluttering structure.

(i) Flutter-based mechanism: In the aeroelastic flutter-based wind energy harvesters, an airfoil is generally attached to the free end of the beam as shown in Figure 10B. When wind flow velocity reaches a sufficient level known as the critical flutter speed, negative damping occurs and divergence of flutter deformation results [67]. The relationship between critical flutter speed and flutter frequency for two degree of beams is approximately expressed by [72]:

(16) $$V_{c}\sim {\left(\frac{YT^{3}}{\rho L^{3}}\right)}^{\frac{1}{2}}$$

(17) $$\omega \sim {\left(\frac{\rho V^{2}}{\rho_{b}T\;L}\right)}^{\frac{1}{2}}$$

where $V_{c}$ is the critical flutter speed, ω is the flutter frequency, ρ and $\rho_{b}$ are the fluid and beam densities respectively, V is the wind speed, *T* is the beam thickness, *L* is the beam length and *Y* denotes Young’s modulus of the beam. Bibo et al. [73] presented a piezoelectric wind energy harvester integrated with an airfoil, the equations governing the motion of this lumped-parameter system can be written as:

(18) $$m_{T}\ddot{h}+m_{w}X_{G}\ddot{\alpha }+C_{h}\dot{h}+K_{h}h-\theta U=L$$

(19) $$I_{\alpha }\ddot{\alpha }+m_{w}X_{G}\ddot{h}+C_{\alpha }\dot{\alpha }+K_{\alpha }\alpha =M$$

(20) $$C_{pz}\dot{U}+\frac{U}{R}+\theta \dot{h}=0$$

where $m_{T}$ is the total mass of the airfoil plus the supporting structure, $m_{w}$ is the airfoil mass alone; $X_{G}$ is the dimensionless distance between the elastic axis and the center of mass, $C_{h}$ and $C_{\alpha }$ are the plunge and pitch structural damping coefficients respectively, and $I_{\alpha }$ is the mass moment of inertia. The linear structural stiffnesses for the plunge and pitch are $K_{h}$ and $K_{\alpha }$ respectively. The electromechanical coupling factor is denoted by θ, while *U* is the generated voltage and *R* is the load resistance. The nonlinear load can be presented by aerodynamic lift *L* and moment *M,* which are equal to [74]:

(21) $$L=\rho V^{2}bSC_{L}\left(\alpha_{eff}-c_{3}\alpha_{eff}^{3}\right),\;M=\left(\frac{b}{2}+a\right)L$$

where *V* is the wind flow velocity, *b* is the half chord length, *S* is the airfoil span, $C_{L}\;$is the aerodynamic lift coefficient, and *c_3_* is a nonlinear parameter derived from wind tunnel tests, $\alpha_{eff}$ is the effective angel of attack and *a* is the elastic axis distance from the mid-chord.

(ii) Galloping-based mechanism: as shown in Figure 10C, when a prismatic object (such as square, D-section, triangle, etc.) is attached to the free end of a flutter, the oscillation proportional to the incoming wind flow can be formed in a plane [75]. The required condition for the galloping oscillations is the derivative of the steady state aerodynamic lift coefficient is negative. Its main difference from the flutter-based mechanism is that it is a one degree-of-freedom system whereas a flutter-based harvester may be a two- or three-degree-of-freedom system. Sirohi et al. [76] developed a wind energy harvester with galloping piezoelectric beam. The proposed device in that study includes an aluminum beam with 90 mm long, 38 mm width and 0.635 mm thickness. Moreover, two piezoelectric sheets with 72.4 mm, 36.2 mm and 0.267 mm in length, width and thickness respectively are bonded to the top and bottom surface of the aluminum beam. Eventually, a rigid wooden bar with 235 mm long and D-shaped cross section with 30 mm diameter is attached to the free end of the beam. This energy harvester was able to generate 1.14 mW power at 4.69 m/s wind speed.

(iii) Flapping-leaf-based mechanism: For this mechanism, a flexible leaf or flag is attached to the free end of the beam as shown in Figure 10D. When wind flow is blowing, the leaf can be moved upwards and downwards to produce vibration. Li et al. [77,78] proposed a flapping-leaf-based wind energy harvesting device that has the potential to extract energy from low wind velocity and irregular flow. This study deployed a piezoelectric stalk (made of polyvinylidene fluoride (PVDF)) as an active part of the wind energy harvester. To increase vibration amplitude, a flexible leaf with 8*8 cm^2^ was attached to the end of this stalk. With a dimension of 72 × 16 × 0.41 mm^3^, the proposed device could generate peak power of approximately 615 μW at 8 m/s wind speed. The required big leaf at the end of the cantilever (stalk) and the generated power amount show that power density of the proposed wind energy harvester is not that high. Moreover, the required large area for flapping operation is one of its major drawbacks.

Numerous methods for predicting and modeling the effect of VIV on the structure are available in the literature. Among them, the phenomenological models based on wake oscillators have high accuracy for representations due to the considered nonlinear factors (e.g., softening and hardening) [79]. Most recently, Dai et al. studied more accurate modeling for the fluctuation lift force and investigated the passive suppression mechanism of the cylinder VIV by means of a nonlinear energy sink [80]. To model the galloping-based harvesters, the quasi-steady approximation method may be used [81]. To improve the applicability of this method, further advancement is still required by devising unsteady representations to specify the galloping force. Thus, the effects of the unsteady wake can be taken into account. Moreover, this study on modeling method should be validated and supported by sufficient experimental measurements.

In the literature, almost all the presented mathematical models for aeroelastic flutter-based wind energy harvesters are based on lumped models, where the flutter is considered as a mass-spring-damper system. The main advantage of this modeling is simplicity for extraction of motion equations, which can be utilized for harvesting performance estimation and structural parameter optimization. However, this model is limited due to lack of parameters to consider piezoelectric characteristics and substrate layers. In addition, the lumped-models cannot present the status of dynamic mode shapes and strain distribution. Therefore, there is still a lot of room for improving such models in the future research to account for nonlinear effects of the piezoelectric materials as well as beam’s inertia and geometric nonlinearities.

**Figure 12:**
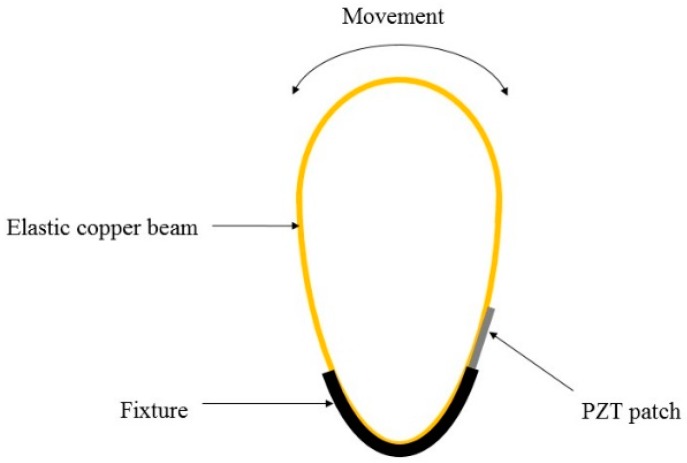
Schematic diagram of the arc shape wind energy harvester.

Compared with the rotational mechanism, the aeroelastic mechanism is able to build up very small wind energy harvesting devices. Since it just needs a narrow ribbon for flapping in proportion with air flow power, the required space for this strip is small. Furthermore, the fabrication of this mechanical mechanism is less complex than the rotational one. However, the major drawback of the aeroelastic mechanism is that its efficient operation only occurs in just one direction although wind flow may have arbitrary directions. Zhao et al. [82] proposed a portable wind energy harvester by using aeroelastic mechanism, which had an arc shape fabricated by a copper plate. The schematic diagram of the proposed energy harvester is illustrated in Figure 12. This study clearly showed that a maximum power could be harvested while wind flow incident was perpendicular to the resonator. To overcome this problem, some techniques such as using funnel have been suggested. 

**Figure 13:**
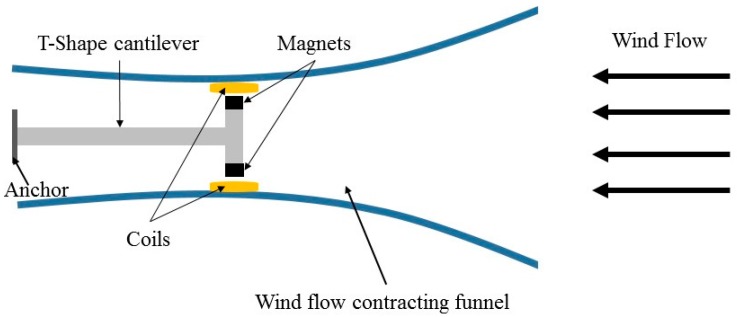
The electromagnetic wind energy harvester with wind flow contracting funnel.

Park et al. [83] proposed a portable wind energy electromagnetic harvester using the aeroelastic mechanism, which consisted of a T-shape cantilever and a magnet attached at the end of the cantilever. As illustrated in Figure 13, two coils were fixed at a location very close to the end of the cantilever. Both the cantilever and coils were located inside a funnel, which had two openings as inlet and outlet for wind flow. The inlet of 4 × 4 inch^2^ and outlet of 2 × 2 inch^2^ with 8 inch distance from each other were patterned. The reported experimental results showed that the funnel could magnify wind speed by approximately 20% while the incident angle was at least 30 degrees. However, utilizing funnel may make the device size larger, which may reduce the portability.

## 4. Dimension of Portable Wind Energy Harvesters

The portable wind energy harvesters may have varied sizes from several micrometers to many centimeters. The reported harvesters from the literature can be classified into two major groups, macro and micro sizes, in terms of physical dimensions. In this paper, we are using the following definition for us to differentiate macro- and micro-scale wind energy harvesters. We consider those devices with any aspect (e.g., length or diameter) greater than 75 mm as the macro-scale wind energy harvesters, while the other ones are categorized as the micro-scale harvesters. In the following sub-sections, we will present each group with a focus on their properties. Moreover, the fabrication methods for the macro- and micro-scale wind energy harvesters will be briefly discussed as well.

### 4.1. Macro-Scale Harvesters

Industry and academia have introduced macro-scale wind energy harvesting devices with dimensions greater than several centimeters. The mechanical aeroelastic mechanism can be fabricated in macro-scale size for portable energy harvesters. Fei et al. proposed a wind-flutter harvester for powering wireless sensors, which encloses a one-meter belt to gain 7 mW electrical energy from 3 m/s wind speed [84]. Matova et al. presented a wind energy harvester by using micromachining piezoelectric on diaphragm of Helmholtz resonator [85]. The proposed device has a neck with length of 2 cm and diameter of 2 cm along with a cavity with length of 17 cm and diameter of 8 cm. The resonator worked in an airflow region from 10 to 15 m/s wind speed. The obtained power was 2 μW at 13 m/s. After Helmholtz structural optimization, the harvested energy was improved to 42.2 μW at 20 m/s. This device may be considered as a portable wind energy harvester, but its level of portability is fairly low. Moreover, its amount of the generated power is also quite small.

Compared to the micro-scale devices, the macro-scale wind energy harvesters have simpler structures. As shown in Equation (2), there is a strong direct relation between the generated power amount and size of wind energy harvesting devices. Therefore, the macro-scale wind energy harvesters can normally offer much larger output power. The rotational mechanism for both piezoelectric- and electromagnetic-based harvesters can be implemented with a small fan. These rotational harvesters [53] normally fall into the macro-scale category due to the large size of their used fans. The rotation of the fans can directly present motion to the rotor part of the electromagnetic-based harvesters [61], while piezoelectric materials can be located on the fans in the piezoelectric-based harvesters so that rotational movement can stress the piezoelectric materials to produce output electrical power [62]. 

The mechanical aeroelastic mechanism can be also fabricated in the macro scale domain for portable wind energy harvesters. The electromagnetic-based harvesters by using cantilever can provide relative motion between active part and stator, where coils or permanent magnets are attached to the end of the free-moving cantilevers for maximum deflection amplitude [86]. In contrast, the piezoelectric technique can also offer an integrated macro-scale wind energy harvester by bonding a ceramic piezoelectric material and a metal ribbon serving as the cantilever. Sirohi et al. [87] proposed an energy harvester with two identical aluminum cantilevers with a dimension of 161 mm × 38 mm × 0.635 mm. The piezoelectric films can be bonded near the root of the cantilevers by using epoxy, hot air plastic welding, or pressure. 

Table 3 summarizes the previously published macro-scale portable wind energy harvesters by listing their geometry sizes, harvested electrical voltages, harvested electrical powers, normalized power densities, power coefficients, wind speeds, utilized power generation techniques, and deployed mechanical mechanisms for wind flow trapping. By default the harvester geometry size is given in the form of *length × width × height*, or diameter (*φ*) and thickness (*t*) if specified. The normalized power density (with unit of μW *×* s/(mm^3^ *×* m)), which is shown in the 5th column, is defined as Equation (3), while the power coefficient, $C_{p}$ defined as Equation (4), is listed in the 6th column of Table 3.

From Table 3, one can observe that the largest harvested voltage can reach up to 30 V, while most of the macro-scale harvesters can only output several volts with the minimum output voltage of 80 mV. The largest output harvested power can reach 171 mW, while the minimum one is only 2 μW. The maximum normalized power density is 5.1 μW × s/(mm^3^ × m) for one electromagnetic generator based on aeroelastic mechanism [88] and the maximum power coefficient is 8.13% for a rotational electromagnetic-based generator [89]. Among these macro-scale wind energy harvesters, the rotational mechanism tends to be less popular in comparison with the aeroelastic mechanism for trapping wind flow. Both electromagnetic and piezoelectric power generation techniques are popular for this group of portable wind energy harvesters. Moreover, a majority of the macro-scale harvesters can provide several milliwatts of output electrical power. 

### 4.2. Micro-Scale Harvesters

**Figure 14:**
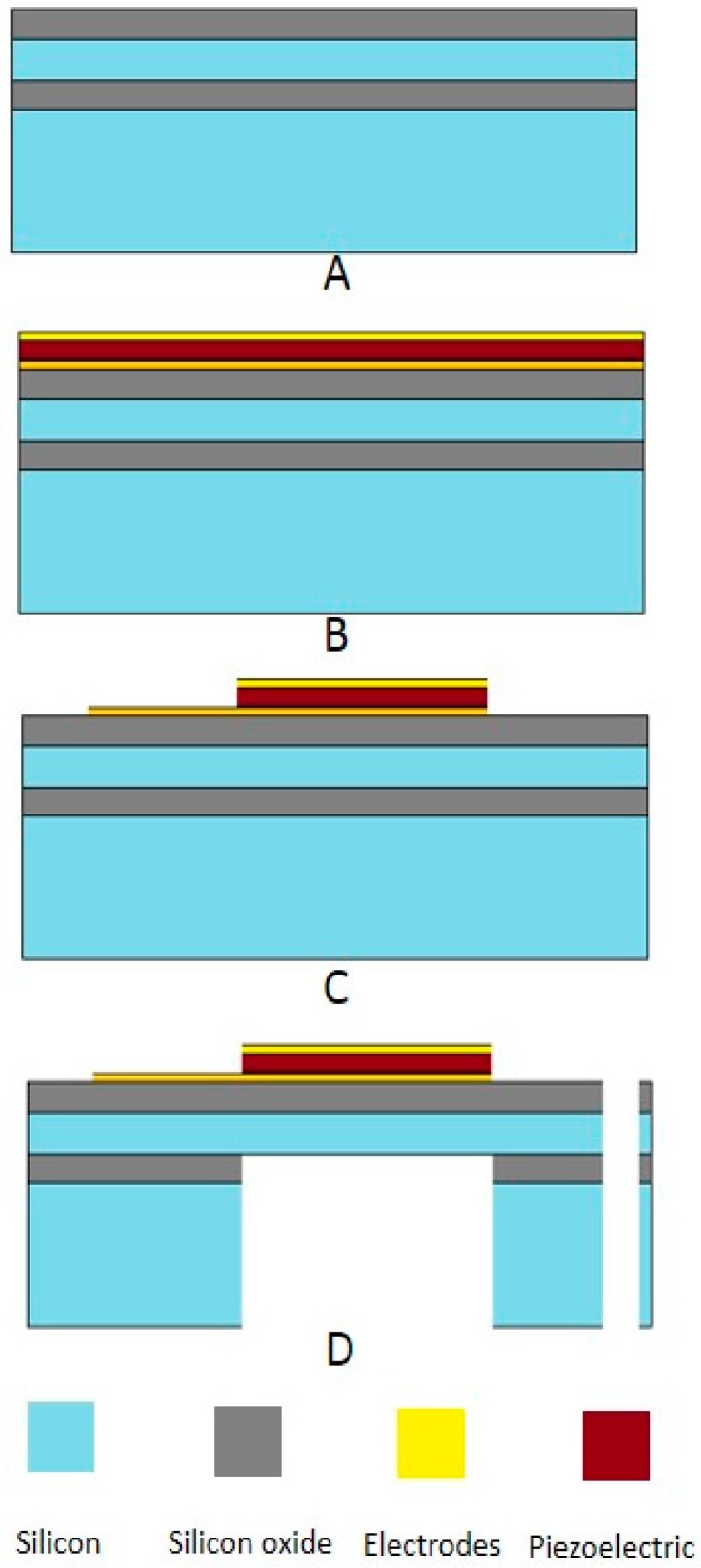
MEMS process fabrication flow. (**A**) Silicon on insulator (SOI) wafer with buried oxide layers; (**B**) Bottom and top electrodes deposited and piezoelectric material sandwiched between them; (**C**) Required pattern on electrodes; (**D**) Backside and frontside silicon etching has been performed with piezoelectric cantilever structure and proof mass released.

Micromachining technology is the key of manufacturing a device in tiny dimension. The cooperation between micro-mechanical parts, which are fabricated by micromachining techniques, and micro-electrical parts offer a MEMS device. Inertial sensors, which can be used for navigation purpose, have always seen their successful MEMS applications for consumer electronics [99]. Wind energy harvesting in micro-scale dimension can be also offered by using this technology. The regular MEMS process flows, such as sputtering, material deposition, etching, etc., can readily build a tiny integrated wind energy harvester with ultra-light weight. Thus, the portability can be significantly improved. Figure 14 illustrates a complete MEMS process flow for manufacturing a piezoelectric-based micro-scale wind energy harvester [100]. Firstly, silicon oxide is grown on silicon wafer (Figure 14A). At the next step, Pt/Ti is deposited as a bottom electrode by DC magnetron. The PZT material, which is commercially available as sol-gel, is deposited. Then on its top, Pt/Ti is deposited again for top electrode as shown in Figure 14B. Afterwards certain required shape can be patterned by etching process to form a whole chip as depicted in Figure 14C. In Figure 14D, the final chip structure of the wind energy harvester after several backside and frontside silicon etching steps is depicted.

Micromachining technology is able to produce micro wires with multiple turns as coils in the electromagnetic-based energy harvesters. Micro-scale electromagnetic energy harvesting based on the aeroelastic mechanism from low-frequency ambient vibration was introduced by Sari et al. [101], who proposed a wideband electromagnetic micro-power harvester by using several cantilevers with varied lengths. The micro wiring was located around the surface of the cantilevers. The fabrication process of the proposed energy harvester required 5 masks for patterning. It had a square shape with one opening in the center for locating the magnet. Although the limit of cantilever length may shrink the amount of the generated power, this effect can be alleviated by increasing the number of cantilevers. Park et al. [102] proposed a micro-electromagnetic harvester for collecting energy from low-frequency ambient vibration. In this study, spiral spring was patterned on silicon wafer by using the bulk micromachining technology. Multi-turned copper micro-coil was manually added and NdFeB magnet as inertial mass was fixed on the spring. As a result of ambient vibration, the spring moved and the magnet would move accordingly. Thus, electrical power could be induced in the copper coil. The total size of the fabricated micro-power harvester was 10 × 10 × 6 mm^3^, which could generate 115.1 μW power and 68.2 mV load voltage when variation was 0.57 g (g = 9.8 m/s^2^) at 54 Hz.

Table 4 summarizes the previously published micro-scale portable wind energy harvesters by listing their geometry sizes, harvested electrical voltages, harvested electrical powers, normalized power densities, power coefficients, wind speeds, power generation techniques, and mechanical mechanisms for wind flow trapping. By default the harvester geometry size is given in the form of *length × width × height*, or diameter (*φ*) and thickness (*t*) if specified. Compared to the macro-scale harvesters, one can read that the micro-scale portable wind energy harvesters normally output less harvested voltage and power. The maximum harvested power is 130 mW and the maximum power coefficient is 9.4% for an electromagnetic wind energy harvester based on the rotational mechanism, which was however measured at high wind speed (11.83 m/s) [103]. The maximum normalized power density is 3.1 μW × s/(mm^3^ × m) for an electrostatic wind energy harvester based on the aeroelastic mechanism. This somehow helps exhibit that the electrostatic power generation technique based on the aeroelastic mechanism is advantageous for the micro-scale wind energy harvesters. Furthermore, the piezoelectric-based power generation technique working with the aeroelastic mechanism for wind flow trapping, such as [104], can be considered as another promising option for the micro-scale wind energy harvesters. Generally the electromagnetic-based wind energy harvesters along with the mechanical rotational mechanism have higher power coefficients than the others. Moreover, both electromagnetic- and piezoelectric-based techniques are popular in the designs of micro-scale wind energy harvesters.

**Figure 15:**
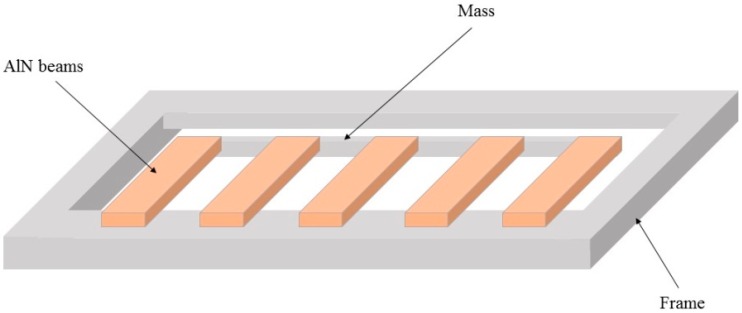
Schematic diagram of 5 AlN cantilevers as an array for energy harvesting.

It tends to be true that the mechanical rotational mechanism for portable wind energy harvesters cannot be achieved by the micromachining technology due to the required fans or blades in the integrated rotation-based wind energy harvesters. Although some parts of the rotational structure, e.g., piezoelectric cantilevers or micro-scale coils, are possible to be fabricated by the micromachining technology, the manufacture of the whole devices seems impractical at the moment. Nevertheless, the advantage of the rotational mechanism in terms of the beneficial power conversion efficiency is definitely obvious compared to the aeroelastic mechanism. On the other hand, a micro-scale wind energy harvesting device may be offered by using piezoelectric generators based on the aeroelastic mechanism thanks to the perfect co-existence capability between piezoelectric materials and micromachining technology. Zhao et al. [116] proposed a micro vibration energy harvester, which was fabricated by using multiple piezoelectric cantilevers with AlN as the piezoelectric material. The film layers were deposited on a silicon wafer by using the magnetron sputtering technique. Eventually the proof mass on each cantilever and other suspending patterns were released by utilizing deep-reactive ion etching. Five piezoelectric cantilevers were used as an array to generate electrical power of 3.315 μW. The schematic of this energy harvester is depicted in Figure 15. Furthermore, some other micro energy harvesters by using micromachining process based on the aeroelastic mechanism have been reported in [50,100].

**Figure 16:**
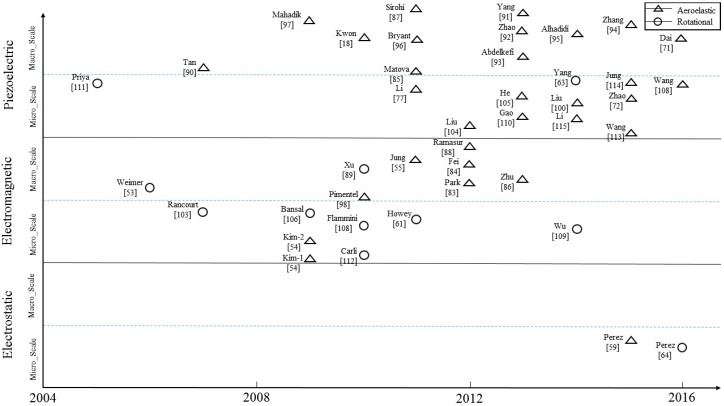
An overview of the surveyed portable wind energy harvesters.

Figure 16 illustrates various features among the portable wind energy harvesters that are reviewed in this paper. It helps exhibit the common features of the energy harvesters along the historical timeline. In this figure, the horizontal axis represents the timeline in years 2004–2016 and the vertical axis covers the information of electrical power generation techniques and device physical dimensions. For each electrical power generation technique, increasing Y amount stands for the increment of the normalized power density. According to Figure 16, the portable wind energy harvesting research and development started with the rotational mechanism, and afterwards the wind energy harvesting based on the aeroelastic mechanism was developed. Since 2013, the piezoelectric technique for the portable wind energy harvesting has become popular. The piezoelectric technique has been widely used in not only macro-scale, but also micro-scale wind energy harvesters. Most recently, the electrostatic technique shows some capability in either aeroelastic or rotational mechanism for wind energy harvesting. Furthermore, by considering the used mechanisms for wind flow trapping in the prior studies, one may observe that the rotational mechanism is less popular than the aeroelastic mechanism, although the former can more effectively extract power from wind flow. Moreover, one can also find that the micro-scale wind energy harvesters have been increasingly developed in the most recent years. In the near future, we may expect that this trend would be continuing to fit for ultra-low-power electronic applications until some commercial micro-scale wind energy harvesting products get available in the market.

## 5. Power Management Systems 

Due to small dimension of the portable energy harvesters, the generated electrical power normally has low or extremely large magnitude, which most of the time is not suitable for practical electronic applications. Therefore, modifying the magnitude is a vital step. Moreover, a direct use of the harvested electrical power is typically impossible due to its AC nature, which needs to be converted to a DC power supply. Therefore, power management systems (PMS) play an important role in conditioning the output voltage or current magnitude and meanwhile providing the DC signals.

The purpose of PMS can be satisfied by a charge pump circuit, also known as a voltage multiplier (VM), whose major configuration is Villard [117]. This voltage multiplier operates with at least two capacitors where one capacitor is charged and the other one is discharged in the first half cycle of the AC input signal and vice versa during the second half cycle. The number of circuit stages specifies the multiplication level of the original signal. Similar to a rectifier circuit, the voltage multiplier outputs a DC voltage, whose power can be simply stored within a super-capacitor. Eventually, any electronic application devices (such as microcontrollers) can draw the required power from the super-capacitor, which can be recharged continuously [118]. 

**Figure 17:**
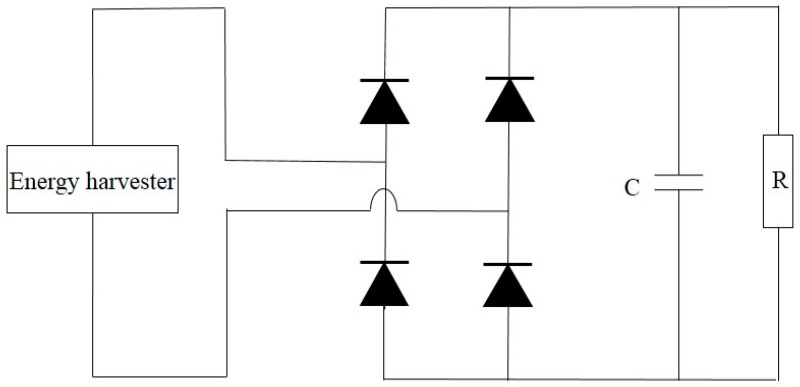
The standard AC-DC circuitry.

The simplest scheme to convert the generated AC signals to DC ones is to use the standard AC-DC circuitry as depicted in Figure 17, which includes a full wave bridge rectifier and a filter capacitor. Four diodes are connected in the bridge configuration to provide unidirectional voltage output. In each cycle only two diodes are forward biased, while the other two diodes are reverse biased. Thus, during each input signal cycle, electrical current is only through one pair of diodes as well as load resistor *R*. In addition, to reduce the ripple on the output voltage, a filter capacitor *C* is used. The experimental validation conducted by Zargarani and Mahmoodi for wind energy harvesting using a piezoelectric flag [119] shows the output power would be reduced as a result of voltage drop across the non-ideal diodes in their forward-biased mode. Since 1N4001 diodes were used with 1V forward bias, a total of 2 V voltage drop across the forward-biased diodes existed in the circuit before reaching the output due to the connection of two series diodes.

**Figure 18:**
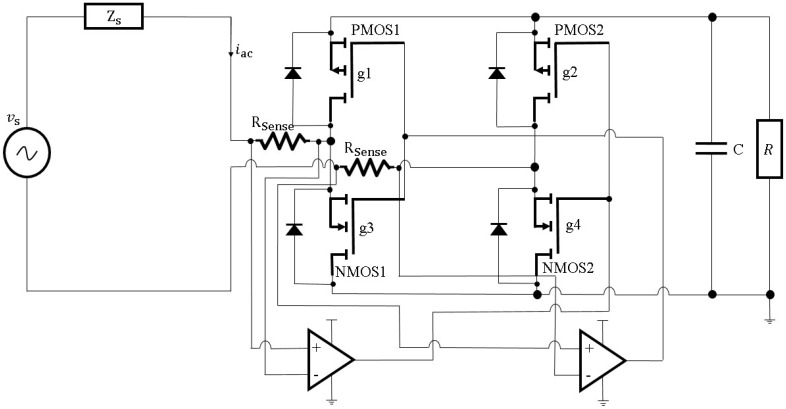
Schematic diagram of the active rectifier bridge.

Tan et al. [120] optimized a power management system for their wind energy harvesting devices. An active rectifier, as shown in Figure 18, with MOSFET transistors instead of the standard diode bridge was developed. The rectifier bridge was composed of a pair of P-type MOSFETs and a pair of N-type MOSFETs. The ON-state voltage drop across each pair of transistors was quite low so that the efficiency could be improved from 40% to 70%. Then a DC-DC boost converter with resistor emulation algorithm took over to perform maximum power point tracking (MPPT) to extract maximum power from wind energy harvesters. As the last stage of the proposed power management system, a super-capacitor was utilized as a power storage unit. The experimental data in this study shows that over the range of wind speed from 2.3 to 8.5 m/s, the efficiency of the MOSFET-based active rectifier is on average 15%–25% higher than the diode-based passive rectifiers.

**Figure 19:**
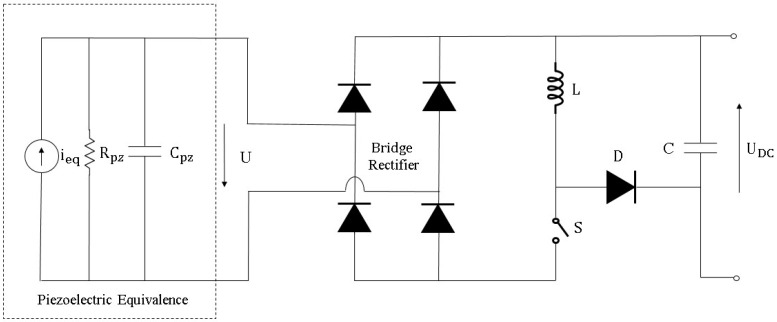
Synchronous charge extraction (SCE) circuitry.

Another configuration for AC-DC converter that has been used for piezoelectric-based energy harvester is synchronous charge extraction (SCE) interface circuitry. As illustrated in Figure 19, the accumulated electrical charge is periodically removed from the energy harvester (C_pz_) and transferred to the load. The energy harvesting with this method consists of two features: firstly extraction phases are synchronized with stimulation forces, secondly the energy harvester itself is considered as an open circuit configuration [121]. A SCE circuit, which has an inductive path in comparison with the standard AC-DC circuit, is composed of a switching component *S*, an inductor *L*, and a diode *D*. When the harvester vibration displacement reaches the extreme position under the stimulation force, *C_pz_*‘s voltage climbs to its peak value. Then switch *S* is turned on so that the energy accumulated in capacitor *C_pz_* is extracted to inductor *L* through *LC* oscillation (composed of *L* and *C_pz_*). After passing a certain time (i.e., a quarter of *LC* oscillation cycle), capacitor voltage *V* drops to 0 and inductor *L*’s current reaches its maximum value. Then, switch *S* is turned off, and inductor *L* freewheels through diode *D* and capacitor *C*. 

Shi et al. studied an efficient self-powered SCE interface circuit for piezoelectric-based energy harvesters [122]. Their work confirms that the stimulation force frequency should be less than *LC* oscillation frequency in order for a SCE circuit to function well. In [123], Zhao and Yang investigated analytical solutions for galloping-based piezoelectric energy harvesters with various interfacing circuits. Their analytical and experimental results show that the power, voltage and vibration displacement are independent of load resistance, which is known as a feature of SCE interface circuits. Moreover, the comparison between standard AC-DC and SCE circuits indicates that the SCE circuit has higher output power than the standard AC-DC one under small electromechanical coupling factor condition of piezoelectric materials.

**Figure 20:**
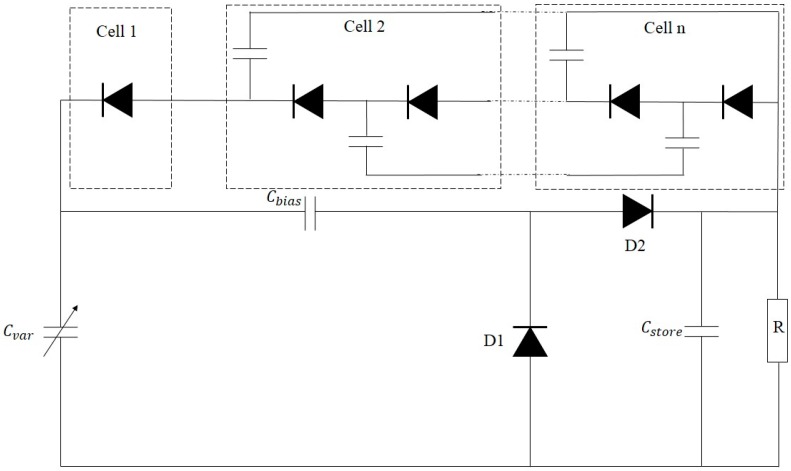
Interface circuit for electrostatic-based energy harvesters.

Wei et al. developed a power management circuit with a simple structure for electrostatic energy harvesters [124]. The proposed circuit, as shown in Figure 20, is only composed of diodes and capacitors. This interfacing circuit includes a source capacitor $C_{var}$, which represents electrostatic generators, a biasing capacitor $C_{bias}$, a storage capacitor $C_{store}$, a pair of rectifier diode D1 and D2, and a voltage multiplier with cells 1, 2 up to *n*. The operation of this circuit consists of two phases. In the first phase, $C_{var}$ decreases due to the discharge through the path of $C_{var}$, $C_{bias}$, D1 and $C_{store}$. In the second phase, $C_{var}$ increases due to the charge with the path of D2, $C_{bias}$ and $C_{var}$. Therefore, the maximum voltage of $C_{var}$ is equal to (*n* + 1) times of $V_{Cstore}$ when $C_{var}$ reaches its minimum value, whereas the minimum voltage of $C_{var}$ is equal to *n* times of $V_{Cstore}$ when $C_{var}$ has its maximum value. The experimental results of this study showed that the efficiencies of over 75% were measured for the harvested power ranging from 13 nW to 75 nW.

Currently some companies, such as Linear Technology [125] and Infinite Power Solution [126], offer integrated kits for converting AC to DC voltage and storing the rectified voltage in super-capacitors. For instance, the low-loss bridge rectifier from Linear Technology has a total voltage drop of about 400 mV under typical piezo-generated currents (~10 μA) and high rectifying conversion efficiency up to 90%. Moreover, typical charge loss of Infinite Power Solution products (e.g., MEC125, MEC120, and MEC) is 2% per year. The stored electrical energy in super-capacitors can be delivered upon the request of power-consuming devices. Thus, the super-capacitors can be repeatedly charged until the electrical power reaches a certain level for practical power supply.

## 6. Conclusions and Discussions

In this paper a comprehensive survey on recent portable wind energy harvesting devices has been conducted. Wind energy harvesters can be categorized into the following three groups in terms of the used power generation techniques: piezoelectric-based harvesters by utilizing the direct effect of piezoelectric materials, electromagnetic-based harvesters by leveraging magnetic flux density changes, and electrostatic-based harvesters by utilizing capacitance variation. In addition, the vibration from wind flow can be collected with the following two mechanical approaches: rotational and aeroelastic mechanisms. The aeroelastic mechanism consists of two groups, vortex-induced vibrations and movement-induced vibrations. Furthermore, considering their physical dimensions, the portable wind energy harvesters are also classified into macro-scale and micro-scale ones. As the generated power amount is strongly dependent on the device size, the macro-scale energy harvesters can typically produce more power than the micro-scale ones.

The current status of research and development exhibits that the aeroelastic mechanism is promising for portable wind energy harvesting mainly thanks to simple structure and ease of fabrication, while the rotational mechanism is quite effective for extracting power from wind flow. The aeroelastic mechanism, which can only operate with good power conversion efficiency when wind flow direction is perpendicular to the structures, should be improved for omnidirectional function by deploying some new symmetric structures. The combination between piezoelectric power generation technique and micromachining fabrication technology can offer a wide range of wind energy harvesters with high portability and reasonable output power. Thus, we expect to see numerous studies in this direction related to piezoelectric materials in the near future.

## Figures and Tables

**Table 1:** The harvestable ambient energy sources.

Ambient Source	Power Density
Solar in outdoor	100 mW/cm^2^
Wind at 4.47 m/s speed	10.4 mW/cm^3^
Thermal at $\Delta T=5{\;}^{{}^{\circ}}C$	60 μW/cm^2^
Water drop with size of 0.35 mL at 3.43 m/s speed	30.67 μW/cm^2^

**Table 2:** Relative electromechanical coupling factors for three different piezoelectric materials.

ZnO	AlN	PZT
0.34	0.47	1

**Table 3:** Macro-scale portable wind energy harvesters (EM stands for electromagnetic and PZ means piezoelectric).

Ref	Size (mm)	Voltage	Peak Power	Normalized Power Density (μW × s/(mm^3^ × m))	Power Coefficient $C_{P}$ (%)	Wind Speed (m/s)	Generation Technique	Mechanical Mechanism
[53]	φ = 76.2	0.15 V	651 μW	-	-	17.5	EM	Rotational
[89]	φ = 76	-	6 mW	-	8.13	3	EM	Rotational
[90]	76.7 × 12.7 × 2.2	8.8 V	155 μW	0.01	0.09	6.7	PZ	Aeroelastic
[55]	φ = 80, t = 10	1.60 V	27.14 mW	0.1	5.7	5.4	EM	Aeroelastic
[85]	φ = 80, t = 170	80 mV	2 μW	1.7 × 10^−7^	2.4 × 10^−5^	14	PZ	Aeroelastic
[71]	90 × 10 × 0.6	12 V	145 μW	0.08	0.63	3.5	PZ	Aeroelastic
[18]	100 × 60 × 30	-	4 mW	0.006	1.73	4	PZ	Aeroelastic
[86]	141 × 100 × 55	3.8 V	573 μW	1.8 × 10^−4^	0.11	4	EM	Aeroelastic
[91]	150 × 30 × 0.6	-	8.4 mW	0.4	0.61	8	PZ	Aeroelastic
[92]	150 × 30 × 1.1	-	6 mW	0.15	0.43	8	PZ	Aeroelastic
[93]	152.4 × 18 × 0.305	-	9.5 μW	0.007	0.12	1.69	PZ	Aeroelastic
[87]	161 ×250 × 0.635	30 V	53 mW	0.4	1.6	5.18	PZ	Aeroelastic
[94]	200 × 15 × 0.8	-	4.5 mW	0.191	0.26	9.8	PZ	Aeroelastic
[83]	203.2 × 50.8 × 50.8	2.1 V	-	-	-	7	EM	Aeroelastic
[95]	209 × 24 × 1	32 V	4 mW	0.073	0.1	11	PZ	Aeroelastic
[96]	254 × 254 × 0.381	-	2.2 mW	0.011	0.011	7.9	PZ	Aeroelastic
[97]	325 × 36.2 × 0.267	30 V	1.14 mW	0.077	0.15	4.69	PZ	Aeroelastic
[88]	490 × 20 ×0.2	6 V	70 mW	5.1	3.46	7	EM	Aeroelastic
[98]	620 ×290 × 750	6 V	171 mW	6.3 × 10^−5^	0.02	20	EM	Aeroelastic
[84]	1000 × 25 × 0.2	3.3 V	7 mW	0.47	1.72	3	EM	Aeroelastic

**Table 4:** Micro-scale wind energy harvesters (EM stands for electromagnetic, PZ means piezoelectric, and ES represents electrostatic).

Ref	Size (mm)	Voltage	Peak Power	Normalized Power Density (μW × s/(mm^3 ^× m))	Power Coefficient $C_{P}\;$(%)	Wind Speed (m/s)	Generation Technique	Mechanical Mechanism
[100]	2 × 1.65 × 0.005	24 mV	34 nW	0.39	0.012	5.2	PZ	Aeroelastic
[105]	3 × 0.3 × 0.008	18.1 mV	3.3 nW	0.03	1.6 × 10^−4^	15.6	PZ	Aeroelastic
[106]	3 × 8 × 0.035	965 mV	2.27 μW	0.17	0.004	16.3	PZ	Aeroelastic
[54]	12 × 12 × 6	81 mV	-	-	-	-	EM	Aeroelastic
[54]	φ = 19, t = 5	4 mV	-	-	-	5	EM	Aeroelastic
[107]	φ = 20	-	4.3 mW	-	2.3	10	EM	Rotational
[108]	23 × 4 × 0.130	1.6 V	0.64 μW	0.004	3.4 × 10^−4^	15	PZ	Aeroelastic
[61]	φ = 32	-	2.5 mW	-	1.51	7	EM	Rotational
[64]	φ = 40, t = 10	-	1.8 mW	0.01	0.24	10	ES	Rotational
[109]	φ = 40	0.6 V	16 mW	-	2.11	9	EM	Rotational
[103]	φ = 42	-	130 mW	-	9.4	11.83	EM	Rotational
[63]	47 × 20 × 0.5	13 V	613 μW	-	-	200 r/min	PZ	Rotational
[110]	75 × 60 × 30	5.2 V	60 mW	0.02	0.39	18	EM	Rotational
[59]	50 × 15 ×0.030	200 V	2.1 mW	3.1	0.29	30	ES	Aeroelastic
[111]	58 × 10 × 0.202	4.3 V	30 μW	0.05	0.06	5	PZ	Aeroelastic
[112]	φ = 53	5 V	7.5 mW	-	6.32	4.47	PZ	Rotational
[82]	60 × 40 × 0.06	34 V	1.73 mW	0.71	0.03	17	PZ	Aeroelastic
[113]	φ = 63, t=41	4.68 V	10 mW	0.02	5.2	4.67	EM	Rotational
[114]	φ = 68, t = 30	70.90 V	9.30 mW	-	-	-	PZ	Aeroelastic
[104]	69 × 37 × 0.24	3.3 V	1 mW	0.81	8.1	2	PZ	Aeroelastic
[77]	72 × 16 × 0.41	3.7 V	615 μW	0.2	0.2	7	PZ	Aeroelastic
[115]	75 × 20 × 0.004	1.2 V	0.98 μW	0.041	0.002	3.9	PZ	Aeroelastic
